# Supplementary material for: Organization and Unconventional Integration of the Mating-Type Loci in Morchella Species
Source: J Fungi (Basel). 2022 Jul 19;8(7):746. doi: 10.3390/jof8070746 (PMC9318749; doi:10.3390/jof8070746)
Supplement: Supplementary file 1 [file jof-08-00746-s001.zip › Supplementary figure 1.pdf]

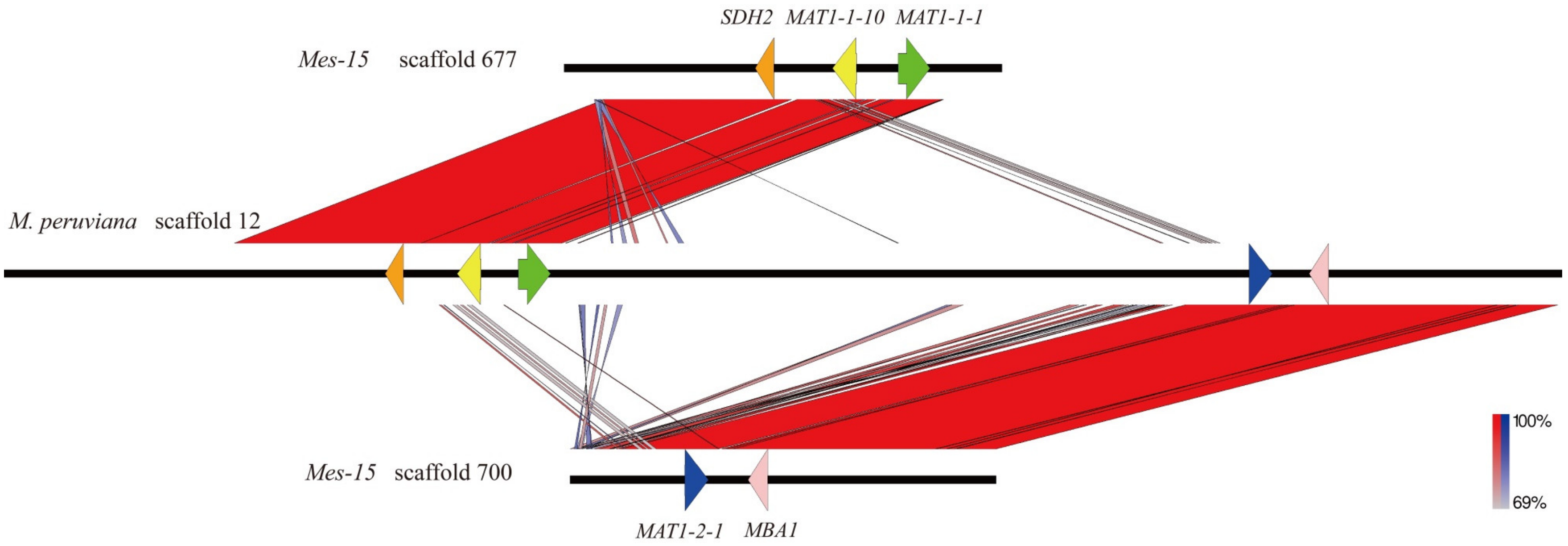

Figure S1. Comparison of the scaffolds including the *MAT* loci between *M. peruviana* and *Mes-15*. Black horizontal lines represent genomic sequences, and color arrows represent coding sequences. Red or blue boxes between genomic sequences indicate pairwise similarity based on BLASTN. Red boxes indicate that both regions are in the same orientation, and blue boxes indicate that the regions are oriented in opposite directions.
